# Supplementary material for: Controlled human hookworm infection remodels plasmacytoid dendritic cells and regulatory T cells towards profiles seen in natural infections in endemic areas
Source: Nat Commun. 2024 Jul 16;15:5960. doi: 10.1038/s41467-024-50313-0 (PMC11252261; doi:10.1038/s41467-024-50313-0)
Supplement: Supplementary file 1 — Supplementary Information [file 41467_2024_50313_MOESM1_ESM.pdf]

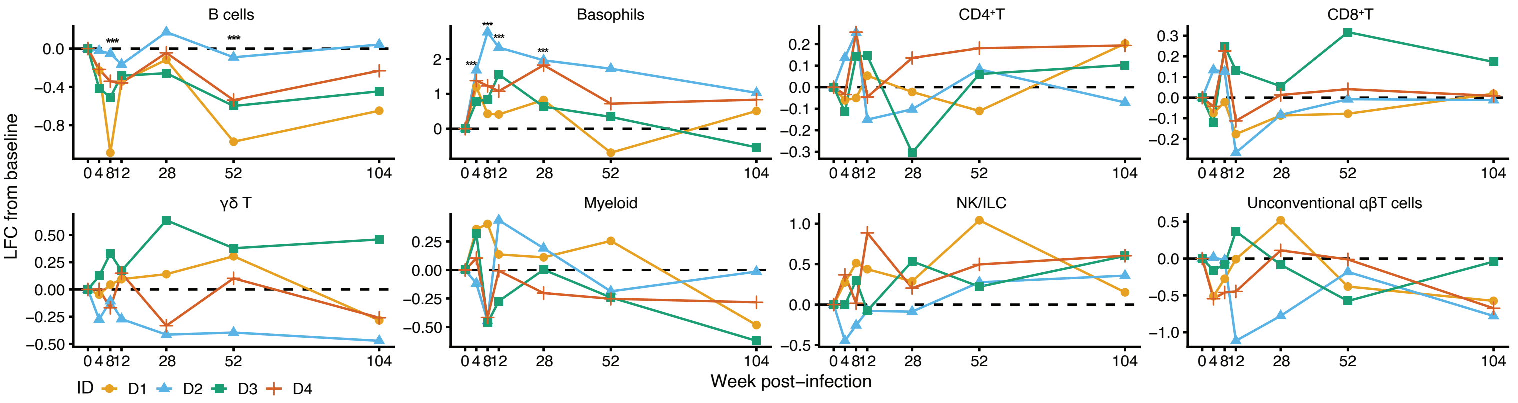

Figure S1**. Changes in lineage frequencies**. Frequencies ofimmune cell lineages were calculated relative to total CD45 $^{+}$ cells and log-fold change (LFC) were calculated relative to the baseline frequencies. Colors and shapes indicated distinct individuals (ID) and paired measurements were indicated with lines connecting data points. Lineage frequencies at post-infection timepoints were tested againstthe baseline (week 0) using binomial linear mixed models. P-values were Dunnet-corrected for multiple comparisons. *** P < 0.001.

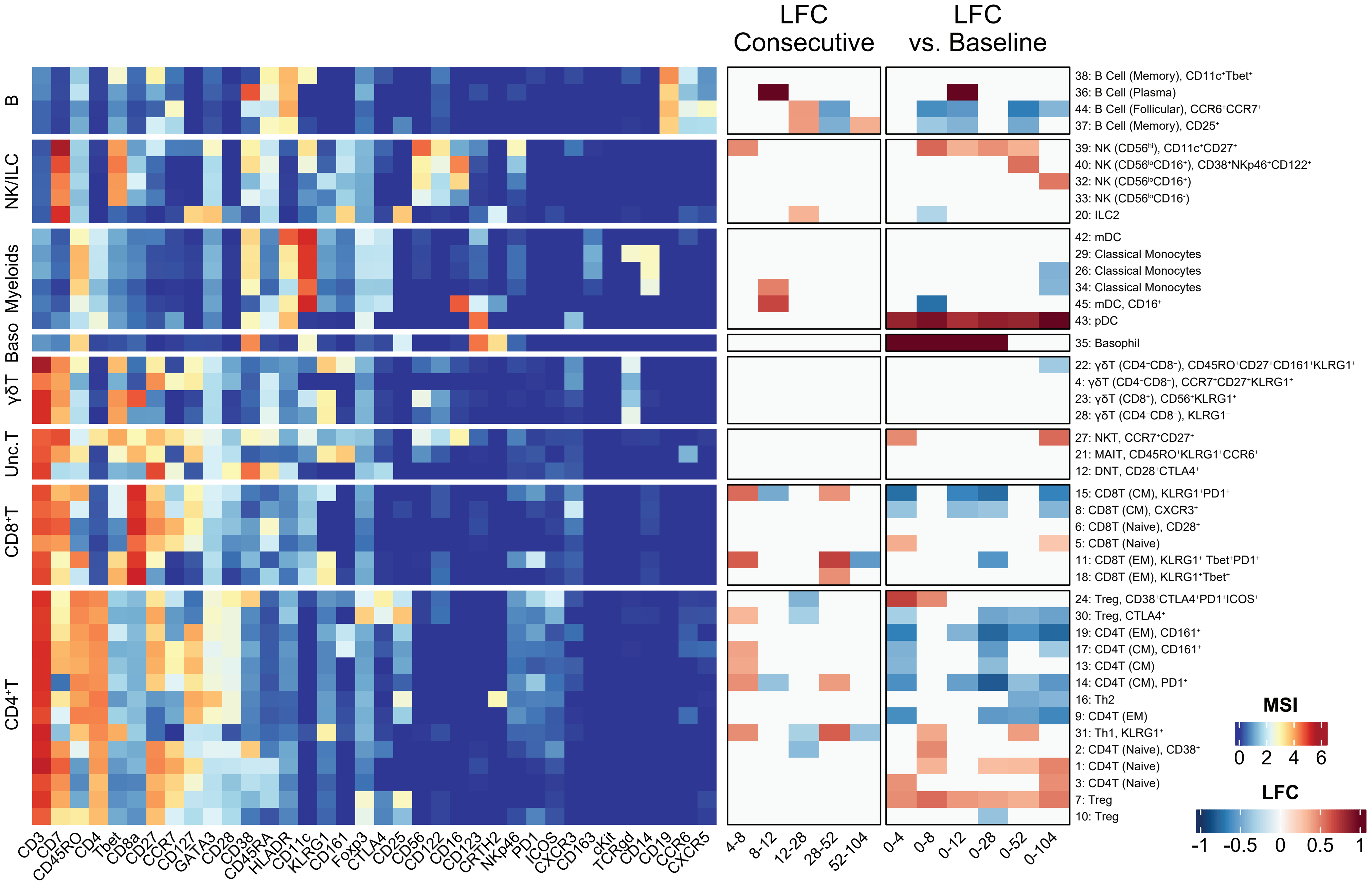

Figure S2. **Summary heatmaps of changes in FlowSOM cluster frequencies stratified by lineage**. Left: heatmap showing median signal intensity (MSI) of marker expression. Right: heatmaps ofl og-fold change (LFC) of cluster frequencies for comparisons against baseline (left panel) or consecutive time points (right panel). FlowSOM cluster frequencies were calculated relative to total CD45+ and frequencies across timepoints compared using binomial linear mixed models. FDR correction was performed using the 'global' FDR method. LFC with FDR >= 0.05 were removed from the heatmap. Unc. T, Unconventional αβ T cells; Baso, Basophils.

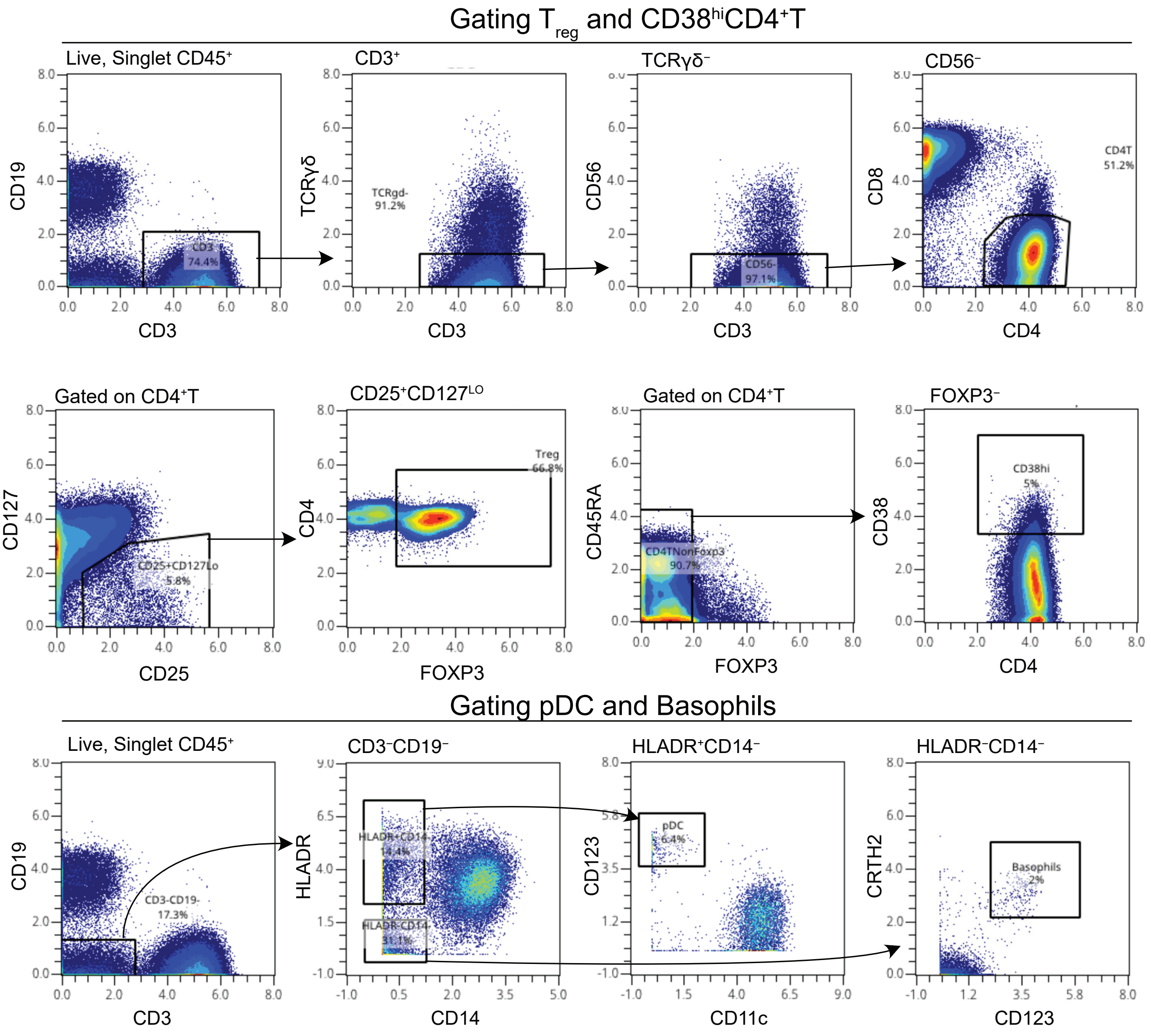

Figure S3**. Gating strategies of specific immune cell subsets from mass cytometry data.**

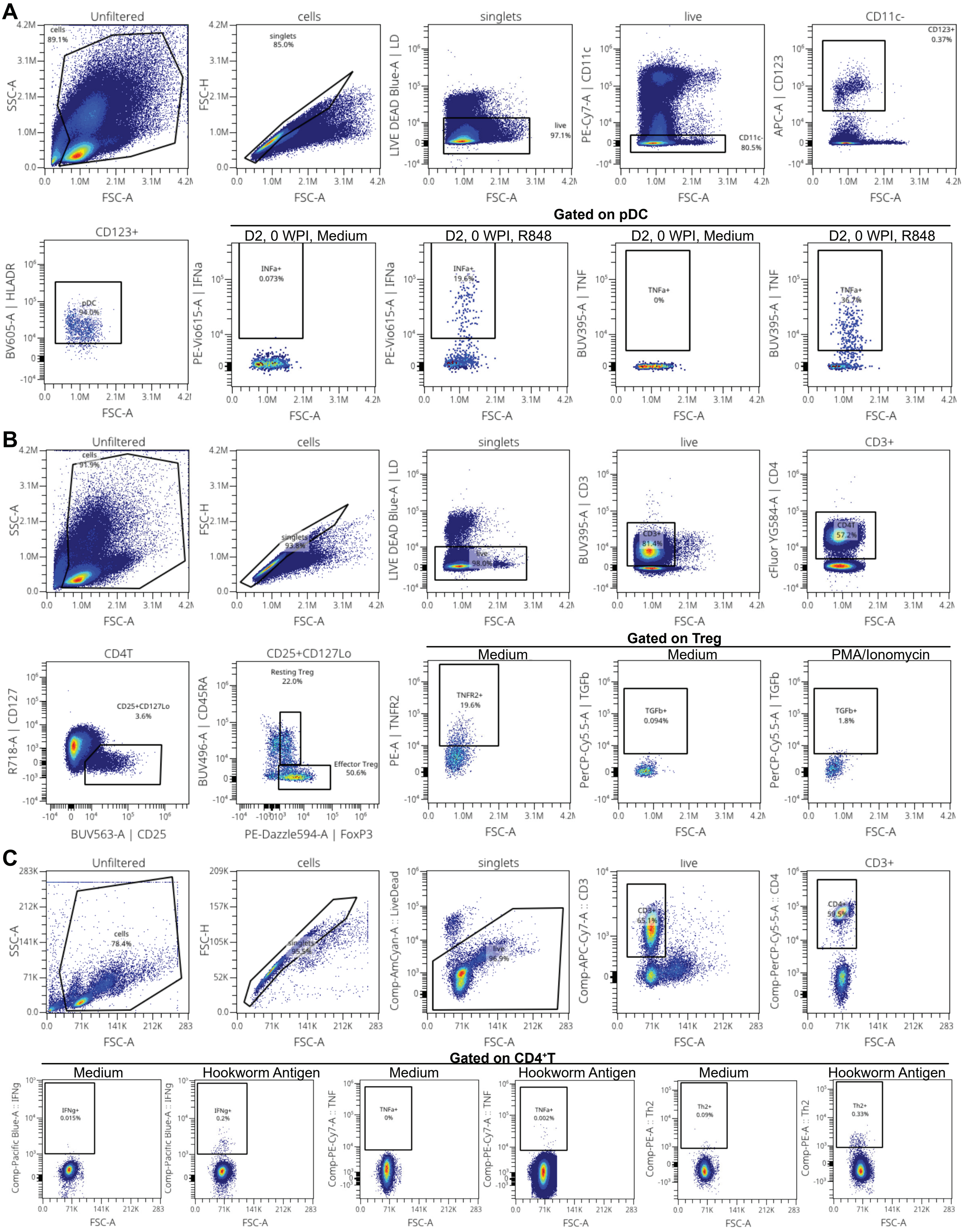

Figure S4. **Gating strategies of flow cytometry intracellular cytokine staining data. (A)** gating of IFNγ- and TNF-producing pDC after TLR7/8 activation with R848. **(B)** gating of regulatory T cells (T_{reg}) expressing TNFR2 and TGF-1 latency- associated peptide (LAP). **(C)** gating of CD4^{+}T cells producing cytokines after stimulation with hookworm crude antigen extract.

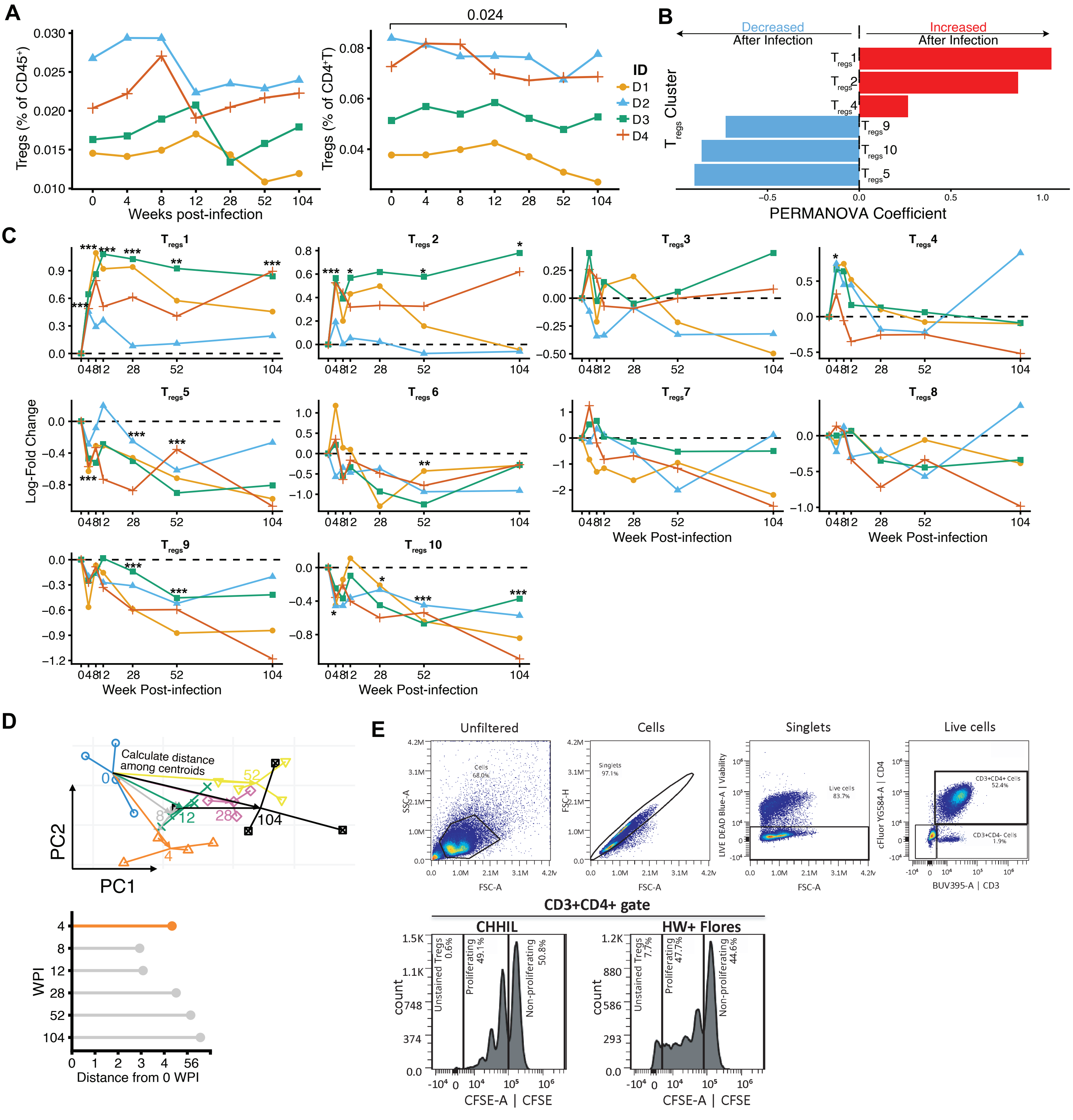

Figure S5. Regulatory T cells. (A) Frequencies of manually gated regulatory T cells (T_{regs}, $CD25^{+-}$ $Foxp3^{+}CD127^{Lo/-}CD4^{+}$T cells) relative to total $CD45^{+}$ (top) or $CD4^{+}$T cells (bottom). P-values were obtained using binomial linear mixed effect models and Dunnet-corrected for multiple comparisons. **(B)** PERMANOVA coefficient of top-3 T_{regs} clusters for each direction of change. T_{regs} cluster frequencies were calculated relative to $CD4^{+}$T cells, within-subject mean-centered, scaled, and then analysed using PERMANOVA. **(C)** Changes in T_{regs} cluster frequencies (relative to $CD4^{+}$T cells) relative to baseline, visualised as log-fold change (LFC). FDR correction was performed using the 'global' FDR method. *** FDR < 0.001, ** FDR < 0.01, * FDR < 0.05. **(D)** Average difference in T_{regs} composition profile compared to baseline (0 WPI). Centroids were defined as the center of samples in each time point in the principal component (PC) space. Euclidean distance between the centroids were then considered as the average distance among Tregs profiles. **(E)** Gating strategy for T_{regs} suppression assay. After gating live, singlet, $CD3^{+}CD4^{+}$T cells, CFSE staining was evaluated to distinguish unstained T_{regs}, proliferating T responders, and non-proliferating T responders. Shown above are the representative CFSE staining in a CHHIL donor and HW^{+} Flores donor.

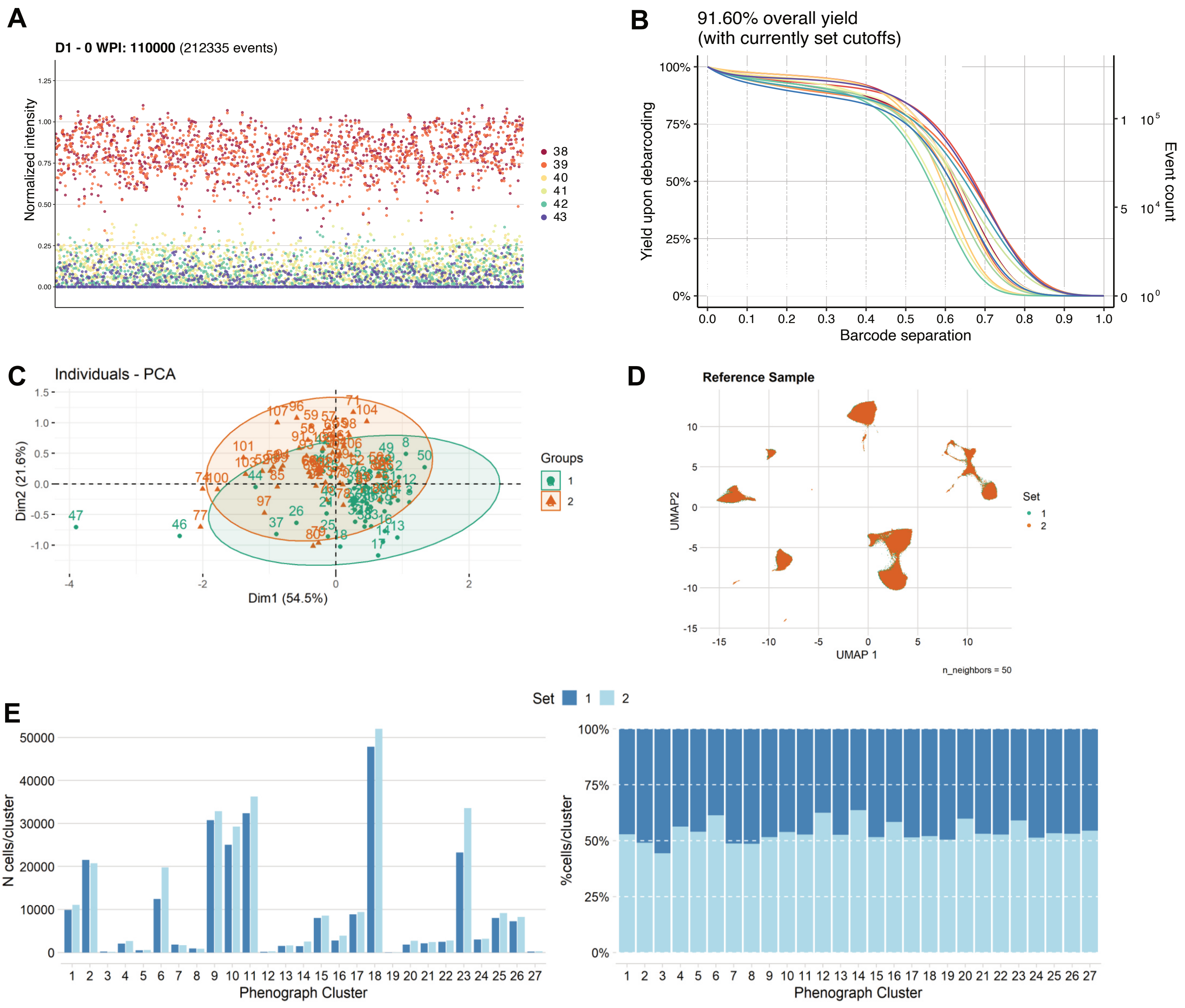

Figure S6. **Mass cytometry data debarcoding and quality control**. **(A)** Representative plot of B_2M barcode signal intensity. **(B)** Estimated cell yield after barcode deconvolution. **(C)** PCA of median signal intensity of all markers from reference samples for the two measurement batches. **(D)** UMAP embedding of downsampled cells (50,000 cells per sample) from the reference samples. **(E)** Relative proportion of cell clusters of the reference sample from the two batches showing comparable proportion of samples from both references in all clusters, indicating lack of or minimal batch effect between measurements. Clusters were obtained using the RPhenograph package with k = 50.

Table S1. **Mass cytometry antibody panel**

Label	Target	Clone	Source	Catalogue	Dilution	Information
^{106}Cd	B_2M	2M2	BioLegend	316302	1/50	Barcoding
^{110}Cd	B_2M	2M2	BioLegend	316302	1/50	Barcoding
^{111}Cd	B_2M	2M2	BioLegend	316302	1/50	Barcoding
^{112}Cd	B_2M	2M2	BioLegend	316302	1/50	Barcoding
^{114}Cd	B_2M	2M2	BioLegend	316302	1/50	Barcoding
^{116}Cd	B_2M	2M2	BioLegend	316302	1/50	Barcoding
^{89}Y	CD45	HI30	Fluidigm	3089003B	1/100	Surface
^{115}In	CD45RA	HI100	BioLegend	304102	1/100	Surface
^{141}Pr	CD196 (CCR6)	G034E3	Fluidigm	3141003A	1/400	Surface
^{142}Nd	CD19	HIB19	Fluidigm	3142001B	1/200	Surface
^{143}Nd	CD117 (c-Kit)	104D2	BioLegend	313202	1/100	Surface
^{145}Nd	CD4	RPA-T4	BioLegend	300541	1/200	Surface
^{146}Nd	CD8a	RPA-T8	Biolegend	301053	1/200	Surface
^{147}Sm	CD183 (CXCR3)	G025H7	BioLegend	353733	1/100	Surface
^{148}Nd	CD14	M5E2	BioLegend	301843	1/100	Surface
^{149}Sm	CD25 (IL-2Ra)	2A3	Fluidigm	3149010B	1/100	Surface
^{150}Nd	CD185 (CXCR5)	J252D4	BioLegend	356902	1/100	Surface
^{151}Eu	CD123	6H6	BioLegend	306027	1/200	Surface
^{152}Sm	TCRγδ	11F2	Fluidigm	3152008B	1/50	Surface
^{153}Eu	CD7	CD7-6B7	BioLegend	343111	1/200	Surface
^{154}Sm	CD163	GHI/61	BioLegend	333602	1/100	Surface
^{155}Gd	CD278 (ICOS)	C398.4A	BioLegend	313502	1/50	Surface
^{156}Gd	CD294 (CRTH2)	BM16	BioLegend	350102	1/100	Surface
^{158}Gd	CD122 (IL-2Rb)	TU27	BioLegend	339015	1/100	Surface
^{159}Tb	CD197 (CCR7)	G043H7	BioLegend	353237	1/100	Surface
^{161}Dy	KLRG1 (MAFA)	REA261	Miltenyi	Special order	1/200	Surface
^{162}Dy	CD11c	Bu15	BioLegend	337221	1/100	Surface
^{164}Dy	CD161	HP-3G10	BioLegend	339919	1/100	Surface
^{165}Ho	CD127 (IL-7Ra)	AO19D5	Fluidigm	3165008B	1/200	Surface
^{167}Er	CD27	O323	BioLegend	302839	1/200	Surface
^{168}Er	HLA-DR	L243	BioLegend	307651	1/200	Surface
^{170}Er	CD3	UCHT1	Fluidigm	3170001B	1/100	Surface
^{171}Yb	CD28	CD28.2	BioLegend	302937	1/100	Surface
^{172}Yb	CD38	HIT2	BioLegend	303535	1/200	Surface
^{173}Yb	CD45RO	UCHL1	BioLegend	304239	1/100	Surface
^{174}Yb	CD335 (NKp46)	92E	BioLegend	331902	1/100	Surface
^{175}Lu	CD279 (PD-1)	EH12.2H7	BioLegend	329941	1/100	Surface
^{176}Yb	CD56	NCAM16.2	Fluidigm	3176008B	1/200	Surface
^{209}BI	CD16	3G8	Fluidigm	3209002B	1/200	Surface
^{169}Tm	GATA3	REA174	Miltenyi	130-108-061	1/50	Intranuclear
^{166}Er	Tbet	4B10	BioLegend	644825	1/100	Intranuclear
^{163}Dy	CD152 (CTLA-4)	BNI3	BioLegend	369602	1/100	Intranuclear
^{160}Gd	Foxp3	PCH101	eBioscience	14-4776-82	1/50	Intranuclear

Table S2. **Mass cytometry FlowSOM cluster annotation.**

Cluster	Lineage	Subset	Annotation
**36**	B cells	Plasma cells	36: B Cell (Plasma)
**37**	B cells	Memory B cells	37: B Cell (Memory), $CD25^+$
**38**	B cells	Memory B cells	38: B Cell (Memory), $CD11c^+$ $Tbet^+$
**44**	B cells	Follicular B cells	44: B Cell (Follicular), $CCR6^+$ $CCR7^+$
**35**	Basophils	Basophil	35: Basophil
**1**	$CD4^+T$	$CD4^+T$ (Naive)	1: $CD4^+T$ (Naive)
**2**	$CD4^+T$	$CD4^+T$ (Naive)	2: $CD4^+T$ (Naive), $CD38^+$
**3**	$CD4^+T$	$CD4^+T$ (Naive)	3: $CD4^+T$ (Naive)
**7**	$CD4^+T$	T_{reg}	7: T_{reg}
**9**	$CD4^+T$	$CD4^+T$ (EM)	9: $CD4^+T$ (EM)
**10**	$CD4^+T$	T_{reg}	10: T_{reg}
**13**	$CD4^+T$	$CD4^+T$ (CM)	13: $CD4^+T$ (CM)
**14**	$CD4^+T$	$CD4^+T$ (CM)	14: $CD4^+T$ (CM), $PD1^+$
**16**	$CD4^+T$	T_H2	16: T_H2
**17**	$CD4^+T$	$CD4^+T$ (CM)	17: $CD4^+T$ (CM), $CD161^+$
**19**	$CD4^+T$	$CD4^+T$ (EM)	19: $CD4^+T$ (EM), $CD161^+$
**24**	$CD4^+T$	T_{reg}	24: T_{reg}, $CD38^+CTLA4^+PD1^+ICOS^+$
**30**	$CD4^+T$	T_{reg}	30: T_{reg}, $CTLA4^+$
**31**	$CD4^+T$	T_H1	31: T_H1, $KLRG1^+$
**5**	$CD8^+T$	$CD8^+T$ (Naive)	5: $CD8^+T$ (Naive)
**6**	$CD8^+T$	$CD8^+T$ (Naive)	6: $CD8^+T$ (Naive), $CD28^+$
**8**	$CD8^+T$	$CD8^+T$ (CM)	8: $CD8^+T$ (CM), $CXCR3^+$
**11**	$CD8^+T$	$CD8^+T$ (EM)	11: $CD8^+T$ (EM), $KLRG1^+Tbet^+PD1^+$
**15**	$CD8^+T$	$CD8^+T$ (CM)	15: $CD8^+T$ (CM), $KLRG1^+PD1^+$
**18**	$CD8^+T$	$CD8^+T$ (EM)	18: $CD8^+T$ (EM), $KLRG1^+Tbet^+$
**4**	$\gamma\delta T$	$\gamma\delta T$ ($CD4^-CD8^-$)	4: $\gamma\delta T$ ($CD4^-CD8^-$), $CCR7^+CD27^+$ $KLRG1^+$
**22**	$\gamma\delta T$	$\gamma\delta T$ ($CD4^-CD8^-$)	22: $\gamma\delta T$ (CD4-CD8-), $CD45RO^+$ $CD27^+$ $CD161^+$ $KLRG1^+$
**23**	$\gamma\delta T$	$\gamma\delta T$ ($CD8^+$)	23: $\gamma\delta T$ ($CD8^+$), $CD56^+KLRG1^+$
**28**	$\gamma\delta T$	$\gamma\delta T$ ($CD4^-CD8^-$)	28: $\gamma\delta T$ ($CD4^-CD8^-$), $KLRG1^-$
**26**	Myeloid	Monocytes ($CD14^+CD16^-$)	26: Classical Monocytes
**29**	Myeloid	Monocytes ($CD14^+CD16^-$)	29: Classical Monocytes
**34**	Myeloid	Monocytes ($CD14^+CD16^-$)	34: Classical Monocytes
**42**	Myeloid	DC (Myeloid)	42: mDC
**43**	Myeloid	DC (Plasmacytoid)	43: pDC
**45**	Myeloid	DC (Myeloid)	45: mDC, $CD16^+$
**20**	NK/ILC	ILC (Type 2)	20: ILC2
**32**	NK/ILC	NK ($CD56^{lo}CD16^+$)	32: NK ($CD56^{lo}$ $CD16^+$)
**33**	NK/ILC	NK ($CD56^{lo}$ $CD16^-$)	33: NK ($CD56^{lo}$ $CD16^-$)
**39**	NK/ILC	NK ($CD56^{hi}$)	39: NK ($CD56^{hi}$), $CD11c^+CD27^+$
**40**	NK/ILC	NK ($CD56^{lo}CD16^+$)	40: NK ($CD56^{lo}$ $CD16^+$), $CD38^+$ $NKp46^+$ $CD122^+$
**12**	Unconventional $\alpha\beta T$ cells	Double negative (DN) T	12: DNT, $CD28^+$ $CTLA4^+$

**21**	Unconventional $\alpha\beta$T cells	MAIT	21: MAIT, CD45RO$^+$ KLRG1$^+$ CCR6$^+$
**27**	Unconventional $\alpha\beta$T cells	NKT	27: NKT, CCR7$^+$ CD27$^+$
**25**	Unassigned	Unassigned	25: Unassigned
**41**	Unassigned	Unassigned	41: Unassigned

FlowSOM clusters are sorted by lineage and cluster number.

Table S3. **Flow Cytometry Antibody Panels.**

Panel	No.	Target	Fluorochrome	Dilution	Clone	Source	Catalogue
1	1	IL-2	FITC	25	5344	BD Biosciences	340448
1	2	IL-4	PE	100	3010.211	BD Biosciences	340451
1	3	IL-5	PE	100	TRFK5	Biolegend	504304
1	4	IL-13	PE	100	JES10-5A2	Biolegend	501903
1	5	CD4	Percp-eF710	400	SK3	eBioscience	9046-0047-025
1	6	TNF	PE-Cy7	1000	MAb11	eBioscience	25-7349
1	7	IL-10	APC	250	JES3-19F1	BD Biosciences	554707
1	8	CD3	APC-eF780	800	UCHT1	eBioscience	47-0038-42
1	9	IFNγ	BV421	1000	4S.B3	Biolegend	502531
2	1	CCR7	BV785	40	G043H7	Biolegend	353230
2	2	CD123	APC	50	AC145	Miltenyi	130-090-901
2	3	BDCA2	PerCPCy5.5	200	201A	BioLegend	354209
2	4	HLA-DR	BUV605	300	G46-6	BD Biosciences	562844
2	5	CD11c	PE-Cy7	400	Bu15	Biolegend	337216
2	6	CD86	PE	400	2331 (FUN-1)	BD Pharmingen	555658
2	7	CD80	V450	500	L307.4	BD Horizon	560442
2	8	IL12	BV421	200	C8.6	BD Horizon	565023
2	9	IFNa	PE-Vio615	50	REA1013	Miltenyi	130-116-875
2	10	TNFa	BUV395	50	MAb11	BD Horizon	563996
2	11	IL1b	FITC	50	JK1B-1	BioLegend	508206
3	1	CD4	cfYG584	750	SK3	CYTEK	SKU R7-20041
3	2	CD25	BUV563	750	2A3	BD Biosciences	612918
3	3	CD45RA	BUV496	750	5H9	BD Biosciences	741182
3	4	CD45RO	BUV805	500	UCHL1	BD Biosciences	748367
3	5	CD3	BUV395	500	UCHT1	BD Biosciences	563546
3	6	PD1	BV750	375	EH12.1	BD Biosciences	747446
3	7	CD127	R718	100	HIL-7R-M21	BD Biosciences	566967
3	8	TNFR2	PE	20	22235	R&D Systems	FAB226P
3	9	GITR	FITC	20	110416	R&D Systems	FAB689F
3	10	IL4	BUV737	750	MP4-25D2	BD Biosciences	612835
3	11	CTLA4	PE-Cy5	300	BNI3	BD Biosciences	555854
3	12	IL10	PerCPef710	250	JES3-9D7	Thermofisher	46-7108-42
3	13	FoxP3	PE Dazzle	75	206D	Biolegend	320126
3	14	TGFb1 (LAP)	PerCP5.5	100	TW4-2F8	BioLegend	349611

Panel 1 was used for the analysis of antigen-specific cytokine-producing CD4+T cells (Fig. 4 B, Table 1)
Panel 2 was used for the analysis of cytokine-producing pDC after R848 stimulation (Fig. 2 G)
Panel 3 was used for functional analysis of regulatory T cells (Fig. 3 J).

Table S4. **Characteristics and helminth infection status of Flores residents.**

Subject Characteristics			PCR (Ct value)#				
**No.**	**Age**	**Sex**	**Hw**	**Al**	**Tt**	**Ss**	**Infection status**
1	18	Male	32.2	31.8	30	Neg	Hw and other
2	18	Male	28.3	Neg	33.4	Neg	Hw and other
3	47	Male	30.2	Neg	Neg	Neg	Hw only
4	55	Male	28.5	30.2	37.9	Neg	Hw and other
5	36	Female	27.8	25.6	28.5	Neg	Hw and other
6	56	Male	38.5	Neg	35.6	Neg	Hw and other
7	28	Female	31.1	Neg	Neg	Neg	Hw only
8	24	Female	34.4	Neg	Neg	Neg	Hw only
9*	18	Male	28	Neg	Neg	Neg	Hw only
10*	50	Male	29	Neg	Neg	Neg	Hw only
11*	31	Female	29	Neg	Neg	Neg	Hw only

Samples were defined as negative by a Ct value of 50. The lower the Ct value, the higher the amount of DNA. * Subject 9, 10, and 11 were included in the analyses corresponding to Fig. 2 G and Fig. 3 J. Flores residents in other analyses correspond to subject 1 to 8. Age of individuals is given in years. Abbreviations: Ct = Cycle threshold; Al = Ascaris lumbricoides; Hw = Hookworm; Tt = Trichuris trichiura; Ss = Strongyloides stercoralis.

Table S5. **Software and algorithms**.

Software and algorithms	Version	Reference
The R Project for Statistical Computing	4.0.2	https://www.r-project.org/
cytofclean	1.0.3	https://github.com/JimboMahoney/cytofclean
ComplexHeatmap	2.12.1	https://bioconductor.org/
CATALYST	1.20.1	https://bioconductor.org/
cyCombine	0.2.13	https://github.com/biosurf/cyCombine
FlowSOM	2.4.0	https://bioconductor.org/
mixOmics	6.20.0	https://bioconductor.org/
vegan	2.6.2	https://cran.r-project.org/
emmeans	1.7.5	https://cran.r-project.org/
lme4	1.1.30	https://cran.r-project.org/
lmerTest	3.1.3	https://cran.r-project.org/
nlme	3.1.159	https://cran.r-project.org/
tidyverse	1.3.2	https://cran.r-project.org/
OMIQ	N/A	www.omiq.ai
FlowJo	v10	https://www.flowjo.com/solutions/flowjo
Cytosplore	2.3.1	https://www.cytosplore.org/
FI-tSNE	1.2.1	https://github.com/KlugerLab/FIt-SNE
