# Supplementary material for: Controlled human hookworm infection remodels plasmacytoid dendritic cells and regulatory T cells towards profiles seen in natural infections in endemic areas
Source: Nat Commun. 2024 Jul 16;15:5960. doi: 10.1038/s41467-024-50313-0 (PMC11252261; doi:10.1038/s41467-024-50313-0)
Supplement: Supplementary file 3 — Description of Additional Supplementary Files [file 41467_2024_50313_MOESM3_ESM.pdf]

Description of Additional Supplementary Files

File Name: Supplementary Data 1
Description: Lineage level differential abundance analysis. P-values and log fold-changes (LFC) were obtained from generalized linear mixed models (GLMM) comparing lineage abundance at post-infection time points (4 to 104 WPI) against baseline (0 WPI). P-values were then FDR-corrected and significant results (FDR < 0.05) are highlighted in green.

File Name: Supplementary Data 2
Description: Cluster-level differential abundance analysis. P-values and log fold-changes (LFC) were obtained from generalized linear mixed models (GLMM) comparing cluster abundance at post-infection time points (4 to 104 WPI) against baseline (0 WPI) or previous time point. Statistically significant comparisons at FDR 0.05 level are highlighted in green. Log-fold changes are highlighted in red or blue to indicate increased or decreased cluster frequencies, respectively. For example, red highlight for 0 to 4 comparison indicate an increase in frequencies from at 4 WPI relative to 0 WPI. For the comparison against baseline, note that comparison between 0 and 4 WPI (0 to 4) were removed due to redundancies with the comparison against previous time point. FDR correction were performed on all P-values. # Correspond to Fig. 3 G, % Correspond to Fig. 2 E, * Correspond to Fig. 2 D.

File Name: Supplementary Data 3
Description: Tregs clusters differential abundance analysis. P-values and log fold-changes (LFC) were obtained from generalized linear mixed models (GLMM) comparing Tregs cluster abundance at post-infection time points (4 to 104 WPI) against baseline (0 WPI) or previous time point. Statistically significant comparisons at FDR 0.05 level are highlighted in green. Log-fold changes are highlighted in red or blue to indicate increased or decreased cluster frequencies, respectively. For example, red highlight for 0 to 4 comparison indicate an increase in frequencies from at 4 WPI relative to 0 WPI. For the comparison against previous time point, note that comparison between 0 and 4 WPI (0 to 4) were removed due to redundancies with the comparison against baseline. FDR correction were performed on all P-values.
