# Supplementary material for: Controlled human hookworm infection remodels plasmacytoid dendritic cells and regulatory T cells towards profiles seen in natural infections in endemic areas
Source: Nat Commun. 2024 Jul 16;15:5960. doi: 10.1038/s41467-024-50313-0 (PMC11252261; doi:10.1038/s41467-024-50313-0)
Supplement: Supplementary file 7 — Reporting Summary [file 41467_2024_50313_MOESM7_ESM.pdf]

nature portfolio

Corresponding author(s): Maria Yazdanbakhsh

Last updated by author(s): Mar 28, 2024

Reporting Summary

Nature Portfolio wishes to improve the reproducibility of the work that we publish. This form provides structure for consistency and transparency in reporting. For further information on Nature Portfolio policies, see our Editorial Policies and the Editorial Policy Checklist.

Statistics

For all statistical analyses, confirm that the following items are present in the figure legend, table legend, main text, or Methods section.

n/a	Confirmed	
☐	☒	The exact sample size (*n*) for each experimental group/condition, given as a discrete number and unit of measurement
☐	☒	A statement on whether measurements were taken from distinct samples or whether the same sample was measured repeatedly
☐	☒	The statistical test(s) used AND whether they are one- or two-sided<br>*Only common tests should be described solely by name; describe more complex techniques in the Methods section.*
☐	☒	A description of all covariates tested
☐	☒	A description of any assumptions or corrections, such as tests of normality and adjustment for multiple comparisons
☐	☒	A full description of the statistical parameters including central tendency (e.g. means) or other basic estimates (e.g. regression coefficient) AND variation (e.g. standard deviation) or associated estimates of uncertainty (e.g. confidence intervals)
☐	☒	For null hypothesis testing, the test statistic (e.g. *F*, *t*, *r*) with confidence intervals, effect sizes, degrees of freedom and *P* value noted<br>*Give P values as exact values whenever suitable.*
☒	☐	For Bayesian analysis, information on the choice of priors and Markov chain Monte Carlo settings
☒	☐	For hierarchical and complex designs, identification of the appropriate level for tests and full reporting of outcomes
☐	☒	Estimates of effect sizes (e.g. Cohen's *d*, Pearson's *r*), indicating how they were calculated

Our web collection on statistics for biologists contains articles on many of the points above.

Software and code

Policy information about availability of computer code

Data collection	Software used include: BD FACSDiva (v8.0.1) for conventional flow cytometry, SpectroFlo (v2.2.03, Cytek Biosciences) for spectral flow cytometry, and Helios (v6.5.358, Standard Biotools) for mass cytometry.
Data analysis	Software and packages used include: R software (v4.0.2), cytofclean R package (v1.0.3, github.com/JimboMahoney/cytofclean), ComplexHeatmap (v2.12.1, R/Bioconductor), CATALYST (v1.20.1, R/Bioconductor), cyCombine (v0.2.13, github.com/biosurf/cyCombine), FlowSOM (v2.4.0, R/Bioconductor), mixOmics (6.20.0, R/Bioconductor), vegan (2.6.2, CRAN), emmeans (1.7.5, CRAN), lme4 (1.1.30, CRAN), lmerTest (3.1.3, CRAN), nlme (3.1.159, CRAN), tidyverse (1.3.2, CRAN), OMIQ (www.omiq.ai), FlowJo (v10), Cytosplore (2.3.1, www.cytosplore.org), FI-tSNE (1.2.1, github.com/KlugerLab/FIt-SNE), miloR (1.10.0, R/Bioconductor). See methods section for complete description.

For manuscripts utilizing custom algorithms or software that are central to the research but not yet described in published literature, software must be made available to editors and reviewers. We strongly encourage code deposition in a community repository (e.g. GitHub). See the Nature Portfolio guidelines for submitting code & software for further information.

Data

Policy information about availability of data

All manuscripts must include a data availability statement. This statement should provide the following information, where applicable:

- Accession codes, unique identifiers, or web links for publicly available datasets
- A description of any restrictions on data availability
- For clinical datasets or third party data, please ensure that the statement adheres to our policy

Mass cytometry dataset from the controlled human hookworm infection trial participant is available in Zenodo as a SingleCellExperiment object (DOI: 10.5281/zenodo.10889855); the repository is currently set in a restricted access and will be adjusted before publication. Further information is available from the corresponding author upon reasonable request.

Research involving human participants, their data, or biological material

Policy information about studies with human participants or human data. See also policy information about sex, gender (identity/presentation), and sexual orientation and race, ethnicity and racism.

Reporting on sex and gender	Our study examined cells from male and female patients. Sex was not considered as a biological variable.
Reporting on race, ethnicity, or other socially relevant groupings	Although the current study involved individuals from different populations (The Netherlands and Indonesia), we focused on the fact that these populations are different in terms of endemicity for hookworm infection.
Population characteristics	Four European, hookworm-naive, volunteers were included in the controlled human hookworm infection in Leiden (CHHIL) trial, of whom 3 were women and 1 was a man, (age range, 19–23 years).<br><br>The Indonesian participants with chronic hookworm infection residing in a hookworm-endemic area were part of the SugarSPIN trial, a household-based cluster-randomized double-blind trial that was conducted in three rural villages in Nangapanda, Ende district of Flores Island (East Nusa Tenggara), Indonesia. See Table S7 for the demographic characteristics of the study population.
Recruitment	For the CHHIL trial: Healthy male and female volunteers aged 18–45 years were recruited in April 2017 and provided written informed consent. Exclusion criteria were a body mass index of <18.0 or >30.0; iron deficiency anemia; positive results of fecal PCR analysis for N. americanus, Ancylostoma duodenale hookworm, or Strongyloides organisms; positive results of serologic analysis for hepatitis B virus, hepatitis C virus, or human immunodeficiency virus; contraindications for the use of albendazole; planned travel to a hookworm-endemic area; incomplete understanding of the study procedures; or any medical condition that could interfere with participation in the trial. See Methods section and Hoogerwerf et al (PMID: 31077279) for more information.<br><br>For the SugarSPIN trial: The study is designed as a household-based cluster-randomized, double-blind trial with two arms for residents living in the three rural villages in Nangapanda. The treatment is provided every three months for a period of 1 year (total 4 rounds) to all household members except children below 2 years of age, while subjects aged 16 and above will undergo clinical and laboratory examination. Subjects with active treatment for diabetes mellitus, serious concomitant disease and pregnancy will be excluded. See Methods section and de Ruiter et al (PMID: 31894102) for more information.
Ethics oversight	The controlled human hookworm infection trial was approved by the local institutional review board (protocol P17.001) and is registered at ClinicalTrials.gov (NCT03126552).<br><br>The SugarSPIN trial was approved by the ethics committee of the Faculty of Medicine, Universitas Indonesia (FKUI) (reference no. 549/H2·F1/ETIK/2013) and filed by the ethics committee of Leiden University Medical Center (LUMC). The trial is registered as a clinical trial (reference no. ISRCTN75636394).

Note that full information on the approval of the study protocol must also be provided in the manuscript.

Field-specific reporting

Please select the one below that is the best fit for your research. If you are not sure, read the appropriate sections before making your selection.

☒ Life sciences ☐ Behavioural & social sciences ☐ Ecological, evolutionary & environmental sciences

For a reference copy of the document with all sections, see nature.com/documents/nr-reporting-summary-flat.pdf

Life sciences study design

All studies must disclose on these points even when the disclosure is negative.

Sample size	The samples used in the current study came from a predesigned clinical trial. The clinical trial was a controlled human infection study, which are generally relatively small due to the novel nature of the study, especially at the time and particularly at the center in which the trial was conducted. No statistical methods were used to determine sample size.

Data exclusions	For the analysis of flow and mass cytometry data, we performed exclusion of doublets, debris, and dead cells. These are common practice in the field. See methods section for complete descriptions.
Replication	The immune profiles and responses were analyzed by mass and flow cytometry, which confirmed and supplemented each other. The study was not replicated.
Randomization	The CHHIL study subjects were not randomized as all individuals underwent the same controlled hookworm infection and treatment regimen. The samples were randomized as much as possible for all assays.
Blinding	Blinding during the clinical part of the hookworm trial was not needed as all study participants received the same controlled hookworm infection and treatment schedule.

Reporting for specific materials, systems and methods

We require information from authors about some types of materials, experimental systems and methods used in many studies. Here, indicate whether each material, system or method listed is relevant to your study. If you are not sure if a list item applies to your research, read the appropriate section before selecting a response.

Materials & experimental systems

n/a	Involved in the study	
☐	☒	Antibodies
☐	☒	Eukaryotic cell lines
☒	☐	Palaeontology and archaeology
☐	☒	Animals and other organisms
☐	☒	Clinical data
☒	☐	Dual use research of concern
☒	☐	Plants

Methods

n/a	Involved in the study	
☒	☐	ChIP-seq
☐	☒	Flow cytometry
☒	☐	MRI-based neuroimaging

Antibodies

Antibodies used

Mass Cytometry Panel: 89Y Anti-Human CD45 Clone HI30 (3089003B; Standard BioTools) (1:100); 115In Anti-Human CD45RA Clone HI100 (304102, BioLegend) (1:100); 141Pr Anti-Human CD196 (CCR6) Clone G034E3 (3141003A, Standard BioTools) (1:400); 142Nd Anti-Human CD19 Clone HIB19 (3142001B, Standard BioTools) (1:200); 143Nd Anti-Human CD117 (c-Kit) Clone 104D2 (313202, BioLegend) (1:100); 145Nd Anti-Human CD4 Clone RPA-T4 (300541, BioLegend) (1:200); 146Nd Anti-Human CD8a Clone RPA-T8 (301053, BioLegend) (1:200); 147Sm Anti-Human CD183 (CXCR3) Clone G025H7 (353733, BioLegend) (1:100); 148Nd Anti-Human CD14 Clone M5E2 (301843, BioLegend) (1:100); 149Sm Anti-Human CD25 (IL-2Ra) Clone 2A3 (3149010B, Standard BioTools) (1:100); 150Nd Anti-Human CD185 (CXCR5) Clone J252D4 (356902, BioLegend) (1:100); 151Eu Anti-Human CD123 Clone 6H6 (306027, BioLegend) (1:200); 152Sm Anti-Human TCRγδ Clone 11F2 (3152008B, Standard BioTools) (1:50); 153Eu Anti-Human CD7 Clone 6B7 (343111, BioLegend) (1:200); 154Sm Anti-Human CD163 Clone GHI:61 (333602, BioLegend) (1:100); 155Gd Anti-Human CD278 (ICOS) Clone C398.4A (313502, BioLegend) (1:50); 156Gd Anti-Human CD294 (CRTH2) Clone BM16 (350102, BioLegend) (1:100); 158Gd Anti-Human CD122 (IL-2Rb) Clone TU27 (339015, BioLegend) (1:100); 159Tb Anti-Human CD197 (CCR7) Clone G043H7 (353237, BioLegend) (1:100); 161Dy Anti-Human KLRG1 (MAFA) Clone REA261 (Special order, Miltenyi) (1:200); 162Dy Anti-Human CD11c Clone Bu15 (337221, BioLegend) (1:100); 164Dy Anti-Human CD161 Clone HP-3G10 (339919, BioLegend) (1:100); 165Ho Anti-Human CD127 (IL-7Ra) Clone AO19D5 (3165008B, Standard BioTools) (1:200); 167Er Anti-Human CD27 Clone O323 (302839, BioLegend) (1:200); 168Er Anti-Human HLA-DR Clone L243 (307651, BioLegend) (1:200); 170Er Anti-Human CD3 Clone UCHT1 (3170001B, Standard BioTools) (1:100); 171Yb Anti-Human CD28 Clone CD28.2 (302937, BioLegend) (1:100); 172Yb Anti-Human CD38 Clone HIT2 (303535, BioLegend) (1:200); 173Yb Anti-Human CD45RO Clone UCHL1 (304239, BioLegend) (1:100); 174Yb Anti-Human CD335 (NKp46) Clone 92E (331902, BioLegend) (1:100); 175Lu Anti-Human CD279 (PD-1) Clone EH12.2H7 (329941, BioLegend) (1:100); 176Yb Anti-Human CD56 Clone NCAM16.2 (3176008B, Standard BioTools) (1:200); 209BI Anti-Human CD16 Clone 3G8 (3209002B, Standard BioTools) (1:200); 169Tm Anti-Human GATA3 Clone REA174 (130-108-061, Miltenyi) (1:50); 166Er Anti-Human Tbet Clone 4B10 (644825, BioLegend) (1:100); 163Dy Anti-Human CD152 (CTLA-4) Clone BNI3 (369602, BioLegend) (1:100); 160Gd Anti-Human FOXP3 PCH101 (14-4776-82, eBioscience) (1:50); 106Cd Anti-Human B2M Clone 2M2 (316302, Biolegend) (1:50); 110Cd Anti-Human B2M Clone 2M2 (316302, Biolegend) (1:50); 111Cd Anti-Human B2M Clone 2M2 (316302, Biolegend) (1:50); 112Cd Anti-Human B2M Clone 2M2 (316302, Biolegend) (1:50); 114Cd Anti-Human B2M Clone 2M2 (316302, Biolegend) (1:50); 116Cd Anti-Human B2M Clone 2M2 (316302, Biolegend) (1:50).

Flow Cytometry Panel 1: FITC Anti-Human IL-2 Clone 5344.111 (RUO (GMP)) (340448, BD Biosciences) (1:25); PE Anti-Human IL-4 Clone 3010.211 (RUO (GMP)) (340451, BD Biosciences) (1:100); PE anti-mouse/human IL-5 Clone TRFK5 (BioLegend) (1:100); PE anti-human IL-13 Clone JES10-5A2 (JES10-5A2, BioLegend) (1:100); PerCP-eFluor 710 Anti-Human CD4 Clone SK3 (9046-0047-025, eBioscience) (1:400); PE-Cy7 Anti-Human TNF alpha Clone MAb11 (25-7349, eBioscience) (1:1000); APC Anti-Human IL-10 Clone JES3-19F1 (RUO) (554707, BD Biosciences) (1:250); APC-eFluor780 Anti-Human CD3 Clone UCHT1 (47-0038-42, eBioscience) (1:800); BV421 Anti-Human IFN gamma (502531, BioLegend) (1:1000).

Flow Cytometry Panel 2: BV785 Anti-Human CD197 (CCR7) Clone G043H7 (353230, BioLegend) (1:40); APC Anti-Human CD123 Clone AC145 (130-090-901, Miltenyi) (1:50); PerCP/Cy5.5 anti-human CD303 (BDCA-2) Clone201A (354209, BioLegend) (1:200); BV605 Anti-Human HLA-DR Clone G46-6 (562844, BD Biosciences) (1:300); PE-Cy7 Anti-Human CD11c Clone Bu15 (337216, BioLegend) (1:400); PE Anti-Human CD86 Clone (2331 (FUN-1)) (555658, BD Biosciences) (1:400); V450 Anti-Human CD80 Clone L307.4 (560442, BD Biosciences) (1:500); BV421 Anti-Human IL-12 (p40/p70) Clone C8.6 (565023, BD Biosciences) (1:200); PE-Vio615 Anti-Human IFN

alpha Clone REA1013 (130-116-875, Miltenyi) (1:50); BUV395 Anti-Human TNF alpha Clone MAb11 (563996, BD Biosciences) (1:50); FITC Anti-Human IL-1 beta (508206, BioLegend) (1:50).

Flow Cytometry Panel 3: cFluorYG584 Anti-Human CD4 Clone SK3(SKU R7-20041, Cytek Biosciences) (1:750); BUV563 Anti-Human CD25 Clone 2A3 (612918, BD Biosciences) (1:750); BUV496 Anti-Human CD45RA Clone 5H9 (741182, BD Biosciences) (1:750); BUV805 Anti-Human CD45RO Clone UCHL1 (748367, BD Biosciences) (1:500); BUV395 Anti-Human CD3 Clone UCHT1 (563546, BD Biosciences) (1:500); BV750 Anti-Human CD279 (PD1) Clone EH12.1 (747446, BD Biosciences) (1:375); R718 Anti-Human CD127 Clone HIL-7R-M21 (566967, BD Biosciences) (1:100); PE Anti-Human TNFR2 Clone 22235 (FAB226P, R&D Systems) (1:20); FITC Anti-Human GITR Clone 110416 (FAB689F, R&D Systems) (1:20); BUV737 Anti-Human IL-4 Clone MP4-25D2 (RUO) (612835, BD Biosciences) (1:750); PE-Cy5 Anti-Human CD152 (CTLA-4) Clone BNI3 (RUO) (555854, BD Biosciences) (1:300); PerCP-eFluor710 Anti-Human IL-10 Clone JES3-9D7 (46-7108-42, Thermofisher) (1:250); PE/Dazzle594 Anti-Human FOXP3 Clone 206D (320126, BioLegend) (1:75); PerCP5.5 Anti-Human LAP (TGF-beta 1) Clone TW4-2F8 (349611, BioLegend) (1:100).

Validation

Well known antibody clones were chosen, titrated, and validated on human peripheral blood mononuclear (PBMC) samples comparable to or from this study. Flurorescence/Mass-minus-one (FMO/MMO) controls, (unstimulated) culture control conditions, and comparison to expression patterns described in literature and on the manufacturer's website were used for validation.

Fluidigm stated that "each lot of conjugated antibody is quality control tested by CyTOF analysis of stained cells using the appropriate positive and negative cell staining and/or activation controls".

Eukaryotic cell lines

Policy information about cell lines and Sex and Gender in Research

Cell line source(s)	HEK-293-TLR4 cells (Cat# 293-htlr4a, Invivogen)
Authentication	*Describe the authentication procedures for each cell line used OR declare that none of the cell lines used were authenticated.*
Mycoplasma contamination	This cell line was tested negative for mycoplasma.
Commonly misidentified lines (See ICLAC register)	*Name any commonly misidentified cell lines used in the study and provide a rationale for their use.*

Animals and other research organisms

Policy information about studies involving animals; ARRIVE guidelines recommended for reporting animal research, and Sex and Gender in Research

Laboratory animals	NIppostrongylus brasiliensis (rodent hookworm)
Wild animals	*Provide details on animals observed in or captured in the field; report species and age where possible. Describe how animals were caught and transported and what happened to captive animals after the study (if killed, explain why and describe method; if released, say where and when) OR state that the study did not involve wild animals.*
Reporting on sex	*Indicate if findings apply to only one sex; describe whether sex was considered in study design, methods used for assigning sex. Provide data disaggregated for sex where this information has been collected in the source data as appropriate; provide overall numbers in this Reporting Summary. Please state if this information has not been collected. Report sex-based analyses where performed, justify reasons for lack of sex-based analysis.*
Field-collected samples	*For laboratory work with field-collected samples, describe all relevant parameters such as housing, maintenance, temperature, photoperiod and end-of-experiment protocol OR state that the study did not involve samples collected from the field.*
Ethics oversight	*Identify the organization(s) that approved or provided guidance on the study protocol, OR state that no ethical approval or guidance was required and explain why not.*

Note that full information on the approval of the study protocol must also be provided in the manuscript.

Clinical data

Policy information about clinical studies
All manuscripts should comply with the ICMJEguidelines for publication of clinical research and a completedCONSORT checklist must be included with all submissions.

Clinical trial registration

The CHHIL trial is registered at ClinicalTrials.gov (NCT03126552). The SugarSPIN trial is registered as a clinical trial (reference no. ISRCTN75636394).

Study protocol

Further information regarding CHHIL study procedure can be obtained in PMID: 31077279.

The SugarSPIN trial study protocol has been published at PMID: 25888525.

Data collection

The CHHIL trial was performed in Leiden University Medical Center, Leiden, the Netherlands. The CHHIL trial participants were

recruited in April 2017, underwent controlled infection in May 2017, and treated in September 2018.

The SugarSPIN trial was conducted in three rural villages in Nangapanda, Indonesia between May 2014 and February 2015.

Outcomes	In the CHHIL trial, the primary outcome is detection of hookworm eggs by faeces microscopy (Kato-Katz) at any week between week 9 to 12 post-infection. The original trial outcomes are also described in clinicaltrials.gov.<br><br>In the SugarSPIN trial, the primary outcome is changes in insulin resistance as assessed by HOMA-IR (Homeostatic Model of Assessment-Insulin Resistance), one year post treatment. See www.isrctn.com/ISRCTN75636394 for more information.

Plants

Seed stocks	No seed stocks were used in the study.
Novel plant genotypes	No plants were analysed in the study.
Authentication	No plants were analysed in the study.

Flow Cytometry

Plots

Confirm that:

- [x] The axis labels state the marker and fluorochrome used (e.g. CD4-FITC).
- [x] The axis scales are clearly visible. Include numbers along axes only for bottom left plot of group (a 'group' is an analysis of identical markers).
- [x] All plots are contour plots with outliers or pseudocolor plots.
- [x] A numerical value for number of cells or percentage (with statistics) is provided.

Methodology

Sample preparation	Cells were thawed, washed in RPMI 1640 supplemented with 100 U/ml penicillin, 100 µg/ml streptomycin, 1 mM pyruvate, 2 mM glutamate, and 10% FCS, and adjusted to a concentration of 5x10^6 cells/ml. Co-stimuli αCD28 (BD Bioscience) and αCD49d (BD Bioscience) were added at a concentration of 1 µg/ml. The cells were then stimulated with 5 µg/ml antigen extract, 200 ng/ml staphylococcal enterotoxin B (SEB; Sigma-Aldrich), and 10% FCS/RPMI for 24 hours at 37° C under 5% CO2. At the last four hours of stimulation, 10 µg/ml brefeldin A (Sigma-Aldrich) was added.<br><br>After stimulation, cells were then washed twice in phosphate-buffered saline (PBS), stained for viable cells with LIVE/DEADTM Fixable Aqua (Thermofisher), and fixed with 1.9% paraformaldehyde (Sigma-Aldrich) in PBS. Subsequently, cells were washed in FACS buffer (0.5% BSA in PBS, Roche, and 2 mM EDTA, Sigma-Aldrich) and then permeabilized with eBioscienceTM Permeabilization Buffer (ThermoFisher). Cells were stained in a 96-well V-bottom plate with 50 µL of flow cytometry panel antibody mixture (Supplementary Table S5) diluted in eBioscience™ permeabilization buffer (ThermoFisher) with 1% human Fc receptor blocker (eBioscience) at 4°C for 30 minutes. The cells were then resuspended in the FACS buffer before acquisition.
Instrument	Stained cells were acquired with a FACSCanto II flow cytometer (BD Biosciences).
Software	The compensation matrix was set using single-stained compensation beads (BDTM CompBead). FCS files were analyzed with the FlowJo v10 software (BD Life Sciences), where positions of gates were guided with fluorescence-minus-one (FMO) or unstimulated controls.
Cell population abundance	N/A
Gating strategy	Gating strategies for flow cytometry data are described in Figure S5.

- [x] Tick this box to confirm that a figure exemplifying the gating strategy is provided in the Supplementary Information.
